# Mental illness and intensification of diabetes medications: an observational cohort study

**DOI:** 10.1186/1472-6963-14-458

**Published:** 2014-10-22

**Authors:** Susan M Frayne, Tyson H Holmes, Eric Berg, Mary K Goldstein, Dan R Berlowitz, Donald R Miller, Leonard M Pogach, Kaajal J Laungani, Tina T Lee, Rudolf Moos

**Affiliations:** Department of Veterans Affairs HSR&D Center for Innovation to Implementation (Ci2i), VA Palo Alto Health Care System, 795 Willow Road (152-MPD), Menlo Park, CA 94025 USA; Division of General Medical Disciplines, Stanford University, Stanford, CA USA; Center for Primary Care and Outcomes Research, Stanford University, Stanford, CA USA; Department of Psychiatry and Behavioral Sciences, Stanford University, Stanford, CA USA; Department of Veterans Affairs Geriatrics Research Education and Clinical Center (GRECC), VA Palo Alto Health Care System, Palo Alto, CA USA; Department of Veterans Affairs Center for Healthcare Organization and Implementation Research, Edith Nourse Rogers Memorial Veterans Hospital, Bedford, MA USA; Department of Health Policy and Management, Boston University School of Public Health, Boston, MA USA; Department of Veterans Affairs, VA New Jersey Health Care System, East Orange, NJ USA; Rutgers University-New Jersey Medical School, Newark, NJ USA

**Keywords:** Psychiatric diagnosis, Diabetes mellitus/therapy, Health care delivery, Hypoglycemic agents/therapeutic use, Veterans, Health services research

## Abstract

**Background:**

Mental health condition (MHC) comorbidity is associated with lower intensity care in multiple clinical scenarios. However, little is known about the effect of MHC upon clinicians’ decisions about intensifying antiglycemic medications in diabetic patients with poor glycemic control. We examined whether delay in intensification of antiglycemic medications in response to an elevated Hemoglobin A1c (HbA1c) value is longer for patients with MHC than for those without MHC, and whether any such effect varies by specific MHC type.

**Methods:**

In this observational study of diabetic Veterans Health Administration (VA) patients on oral antiglycemics with poor glycemic control (HbA1c ≥8) (N =52,526) identified from national VA databases, we applied Cox regression analysis to examine time to intensification of antiglycemics after an elevated HbA1c value in 2003–2004, by MHC status.

**Results:**

Those with MHC were no less likely to receive intensification: adjusted Hazard Ratio [95% CI] 0.99 [0.96-1.03], 1.13 [1.04-1.23], and 1.12 [1.07-1.18] at 0–14, 15–30 and 31–180 days, respectively. However, patients with substance use disorders were less likely than those without substance use disorders to receive intensification in the first two weeks following a high HbA1c, adjusted Hazard Ratio 0.89 [0.81-0.97], controlling for sex, age, medical comorbidity, other specific MHCs, and index HbA1c value.

**Conclusions:**

For most MHCs, diabetic patients with MHC in the VA health care system do not appear to receive less aggressive antiglycemic management. However, the subgroup with substance use disorders does appear to have excess likelihood of non-intensification; interventions targeting this high risk subgroup merit attention.

**Electronic supplementary material:**

The online version of this article (doi:10.1186/1472-6963-14-458) contains supplementary material, which is available to authorized users.

## Background

The drive to “cross the quality chasm” [1] toward better health care for all Americans has drawn attention to variability in processes of health care as a contributor to variability in outcomes [2]. Adherence to process of care standards for diabetes [3] (such as guidelines regarding maintenance of good glycemic control) can reduce the toll of diabetes on health [4, 5]. However, clinicians do not consistently intensify antiglycemic medications in those with poor glycemic control [6]. While sometimes appropriate, [7] causes of non-intensification require inquiry. Mental health condition (MHC) comorbidity could be one cause.

There are several reasons to anticipate that clinicians might respond less aggressively to poor glycemic control in patients with MHC. Adherence concerns, communication barriers caused by clinical manifestations of the MHC, time constraints posed by clinical complexity, or bias towards patients perceived as “difficult” could foster non-intensification [8]. Indeed, MHC-related quality gaps have been documented in diabetes management related to monitoring of the patient, level of glycemic control, or *receipt* of antiglycemic medications [9–14]. However, we are aware of only one prior study that examines effect of MHC upon clinicians’ decisions about *intensification* of antiglycemic medications following an elevated Hemoglobin A1c (HbA1c) value [15]. It is important to determine if decisions about whether to intensify antiglycemic medications contribute to the previously documented tendency for patients with MHC to have disproportionately poor glycemic control [9, 16].

The Veterans Health Administration (VA) is an excellent setting in which to examine this issue. It is the largest integrated healthcare system in the United States, with comprehensive electronic medical records. Mental illness is more common in VA than in the general U.S. population, [17–21] as is diabetes [22]. VA’s performance on diabetes quality measures has improved substantially since tracking began in 1995 [23]. However, individual-level variability persists in glycemic control (measured with HbA1c tests), putting patients at risk of adverse diabetes outcomes. MHC-related differences in intensity of diabetes care could contribute to such variability.

We studied a national cohort of diabetic patients treated with oral antiglycemic agents in VA, asking:

1. After an elevated HbA1c value (poor glycemic control), is delay in antiglycemic medication intensification longer for patients with MHC than for those without MHC?
2. Does any such effect vary by MHC type?

## Methods

### Overview and data sources

In this observational study, the study cohort was derived from the Diabetes Epidemiology Cohorts (DEpiC) database, a validated, cumulative census of all diabetic VA patients [22]. Data came from VA’s National Patient Care Database (NPCD) and Decision Support System (DSS).

We examined differences in antiglycemic intensification for patients with versus without MHC managed with oral antiglycemics who had suboptimal glycemic control. We identified an elevated HbA1c value (“index HbA1c”) occurring during a one-year Observation Interval (OI) from April 1, 2003 through March 31, 2004, and examined intensifications of antiglycemics in the 180 days following the index HbA1c, i.e., in the period ending as late as September 30, 2004. This study was approved by Stanford University’s Administrative Panels for the Protection of Human Subjects.

### Study cohort

See Table 1 for details of cohort construction. DEpiC identified veterans as having diabetes as of the first day of fiscal year 2003 based on receipt of VA antiglycemic treatment in the prior year, or based on at least two instances of VA/Medicare International Classification of Diseases, Ninth Revision, Clinical Modification (ICD-9-CM) diagnosis codes (250.00-250.93, 357.2, 362.0-362.02) in the prior two years [22]. The study cohort was drawn from DEpiC members who received oral antiglycemics from VA at least once during the 6 months pre-OI, used VA outpatient care in the prior year, and were alive on the first day of the OI. We excluded institutionalized patients (the focus was upon outpatient diabetes management) and patients with serious conditions likely to alter goals of care (the focus was upon patients eligible for routine intensification). For complete lab data capture, we excluded patients whose home VA facility did not submit HbA1c data consistently to DSS, the central data repository. This step retained data for 125 facilities (86%) and retained nearly identical proportions of patients with and without MHC.

**Table 1:** **Construction of study cohort**

Sample size n	Criterion	MHC Yes	MHC No
		n***(%)***	n***(%)***
440,953	**Diabetic veteran VA outpatient treated with oral antiglycemic** (during the 6 months prior to the OI), who was alive at start of OI and for whom MHC status can be determined	80,798	360,155
	**↓**		
427,335	VA outpatient use in OI*	80,745	346,590
		*100%*	*96%*
	**↓**		
426,605	Non-institutionalized in OI^†^	80,344	346,261
		*100%*	*100%*
	**↓**		
426,454	No data problems^‡^	80,323	346,131
		*100%*	*100%*
	**↓**		
366,066	No conditions likely to alter goals of care^§^	67,099	298,967
		*84%*	*86%*
	**↓**		
315,063	Patient’s home facility submitted usable HbA1c lab data to central data repository	57,309	257,754
		*85%*	*86%*
	**↓**		
269,692	Had at least one HbA1c test completed during the OI	51,582	218,110
		*90%*	*85%*
	**↓**		
	**Poor glycemic control:** Had at least one HbA1c ≥8 during OI eligible to serve as an index HbA1c:	20,803	71,572
		*36%*	*28%*
	(1) no antiglycemic intensification in the 3 months prior to the HbA1c test, AND	18,802	65,458
		*90%*	*91%*
	(2) no hospitalization in the 3 months prior to the HbA1c test, AND	18,197	64,670
		*97%*	*99%*
	(3) not occurring on a hospital admission day, AND	18,180	64,629
		*100%*	*100%*
58,364	(4) no insulin prescribed in the 6 months prior to the HbA1c test	11,581	46,783
		*64%*	*72%*
	**↓**		
52,526	**Final analytic cohort:** Testing data set (90% random sample)	10,422	42,104
		*90%*	*90%*

***Legend:*** This table shows sample size at each step of cohort construction, overall and for those with/ without Mental Health Conditions; % reported in a row refers to % of prior row remaining after applying the inclusion criterion listed in current row.

*Utilization refers to VA face-to-face outpatient care of any type; telephone, laboratory or radiology encounters did not qualify as face-to-face. Note that percentages in each row use the number in the prior row as denominator.

^†^Did not spend more than half the year in a VA inpatient or long-term care setting.

^‡^Data quality issues such that unique identifier (scrambled social security number), date of birth or vital status was indeterminate.

^§^End-stage renal disease, end-stage liver disease, cancer, stroke or dementia diagnosis occurring in the two years prior to the observation interval.

*Abbreviations:* *OI,* Observation Interval (April 1, 2003 through March 31, 2004); *MHC,* Mental Health Condition; *HbA1c,* Hemoglobin A1c; *VA,* Veterans Health Administration.

Finally, among patients with at least one HbA1c test completed during the OI, we identified patients eligible for intensification as those with at least one “qualifying” HbA1c value ≥8.0 [15, 24] during the OI. An elevated HbA1c value was considered to be a qualifying value if no antiglycemic intensification had occurred in the prior 3 months (since a clinician might not elect to intensify if treatment was changed recently) and if no hospitalization had occurred in the prior 3 months (since a clinician might not intensify if the value reflected transient effects of an acute inter-current illness or if treatment was altered during an inpatient stay) and if the HbA1c test was not obtained on a hospital admission day (for a similar reason). In addition, a HbA1c test was only considered to be a qualifying value if it was not preceded by any insulin prescription in the prior six months. The rationale for this additional requirement was that modifications to insulin dose sometimes are communicated verbally and not recorded, so intensifications in insulin dose cannot be detected reliably. The cohort was therefore composed of patients managed exclusively on oral agents at baseline.

Like others, [15, 24] we selected a threshold of 8.0 for HbA1c. In the calendar period being examined, VA guidelines recommended a risk stratification approach with targets varying based upon comorbidities and life expectancy [25]. The goal of this study was not to assess quality of diabetes care in VA, for which a lower HbA1c threshold would apply to many patients with diabetes. Instead the goal was to examine *differences* in management as a function of MHC status *among patients having a clear indication for intensification*, i.e., in patients not meeting even the conservative threshold of HbA1c <8.0, which would apply to most individuals with diabetes.

Among the 66,798 patients meeting all these criteria, we excluded the 12.6% with indeterminate MHC status (n = 8,434; MHC Indeterminate group not shown in Table 1), defined below. There were thus 58,364 patients who met all study criteria. For each of them, we randomly selected one eligible high HbA1c value within the OI as the “index HbA1c”, with the intent to identify intensification occurring in the 180 days following that index HbA1c. After reserving a randomly-selected 10% of patients for exploratory, model development analyses, the remaining 90% represented the final analytic cohort (n = 52,526; 10,422 MHC Yes and 42,104 MHC No).

### Independent variable: MHC

We used the Agency for Healthcare Research and Quality’s Clinical Classifications Software, with minor modifications, to map ICD-9-CM codes uniquely to ten specific MHCs: depressive disorders, posttraumatic stress disorder (PTSD), other anxiety disorders, adjustment disorders, psychotic disorders, bipolar disorders, substance use disorders, personality/conduct/impulse control disorders, psychogenic disorders, and other mental health conditions. ICD-9-CM code specifications are provided in the Additional file 1. A patient was considered “MHC Yes” if he/she had at least one instance of an MHC ICD-9-CM code in the two years pre-OI, plus a confirmatory code during the OI. This ensured MHC was present before the index HbA1c and ongoing during the OI; requiring the confirmatory code improves robustness of the MHC measure [26]. Patients with no MHC ICD-9-CM in the three-year period were “MHC No.” All others (i.e., those with an MHC ICD-9-CM code in the two years pre-OI *or* during the one year OI, but not both) were “MHC Indeterminate” and were excluded. This allowed direct comparisons between two sharply delineated groups (MHC Yes versus MHC No), enhancing specificity of MHC status classification.

Separate indicator variables were created for each specific MHC. For example, a patient was considered to have “depressive disorder Yes” if he/she had at least one instance of a depressive disorder ICD-9-CM code at baseline and at least one confirmatory code during the OI (versus no instance of depressive disorder in the three-year period). Since an individual could have more than one specific MHC, the same patient could have had a positive indicator variable for another MHC such as PTSD as well.

### Dependent variable: intensification

An intensification was considered to have occurred during the 180 days following the index HbA1c if (a) total daily dose of oral antiglycemic medication (unit dose × quantity issued ÷ days supply) was higher than total daily dose at the time of the most recent prior prescription, (b) a new oral antiglycemic was issued, or (c) insulin was issued, based on VA pharmacy data. (Note: While *changes* to insulin dose may not be detected reliably in pharmacy data, such *new* insulin prescriptions should be.) An oral antiglycemic was “new” if time from end date of the most recent prior prescription of that medication to start of the next prescription of that medication exceeded 6 months: issuing a medication—even at the same or lower dose—following a lapse in therapy indicates active management efforts by the clinician. We linked each index high HbA1c value date to the dates of the most recent prior intensification and the most recent subsequent intensification, if any.

### Competing variables/censoring variables

Following the index high HbA1c, a subsequent HbA1c <8.0 was treated as a competing event [27]. VA hospitalizations (admission dates), death, and no event prior to end of OI were treated as right-censoring.

### Other variables

Age, sex and race/ethnicity came from NPCD. Physical Comorbidity Index is a count of 36 common, non-psychiatric medical conditions developed for VA outpatient case mix adjustment [28]. This, and variables for specific macrovascular or microvascular conditions, were derived from ICD-9-CM codes in the two years pre-OI. Count of primary care visits in the year pre-OI was identified using clinic type codes.

### Analysis

#### Descriptive cohort characteristics

In the analytic cohort, we calculated sample means or percentages for sociodemographic characteristics, health status, and index HbA1c value, for patients with any MHC and for those with no MHC.

#### Bivariate associations: MHC status and intensification or first event type

Using cross-tabulations, we summarized associations between MHC status (yes/no) and intensification within 14 days or within 30 days post index HbA1c, and between MHC status and each of the following as a *first* event during the OI: intensification, HbA1c <8, hospitalization, death and none of these.

#### Main analyses: MHC status and intensification

In developing our model, we used the 10% random sub-sample to assess whether assumptions of Cox regression were met [27]. Then, in the 90% random sub-sample, we used the fitted Cox regression model to test for association between time to intensification (i.e., days from the date of the index elevated HbA1c test to the date that antiglycemic intensification first occurred) and MHC status (MHC Yes versus MHC No). Our model treated hospitalization, death and end of the OI without an event as right censorings. The model controlled for possible confounders, selected *a priori* based on the literature: *sociodemographic characteristics* of age and sex (race/ethnicity was not included due to missingness), *health status* (Physical Comorbidity Index and macrovascular/microvascular complications of diabetes), *glycemic severity* (index HbA1c value), and *facility level clustering* (125 facilities as fixed effects using 124 dummy variables). With the large sample size (n = 52,526), statistical power was more than adequate for detecting clinically meaningful effect sizes.

To meet the linearity assumption, we conducted logarithmic transformation of the Physical Comorbidity Index. To meet the proportional hazards assumption for MHC status, age and microvascular complications, we performed interval-specific analyses, [29] dividing the 180 days following the index HbA1c into three periods: 0–14, 15–30, and 31–180 days post index HbA1c. These periods are clinically meaningful: allowing time for the clinician to receive the lab result, it would be desirable for clinicians to respond to an abnormal lab promptly within two weeks (consistent with the 0–14 day interval), but if an appointment needed to be scheduled to review the results, it could have taken a month to bring the patient in to the office to discuss treatment options (consistent with the 15–30 day interval). Intensifications occurring more than a month after the elevated HbA1c may have been occurring at routine visits not scheduled for the specific purpose of reviewing the test result. We fit the model separately to all patients who had not yet experienced an event during each of these three sequential time periods. In the first two time periods, we treated a subsequent HbA1c <8.0 as a right censoring event, due to the small number of such events (0.1% and 0.2%, respectively). In the last model (31–180 days), we treated a subsequent below-threshold HbA1c value as a competing event; we postulated that once HbA1c <8.0 occurred, a search for subsequent intensification was no longer necessary. For all models, attainment of the end of the time period of interest without an event was treated as an administrative right censoring event.

Reported parameter estimates are from the fit to the 90% sample. From the covariate-adjusted Cox regression models, primary parameter values of interest were hazard ratios (HR), estimated for intensification as a function of MHC status. We also estimated cumulative incidence functions, [30] separately by MHC status (yes/no) and time period (0–14, 15–30, 31–180 days), with all covariates set to their sample means. Cumulative incidence functions were estimated for the below-threshold HbA1c value competing event for 31–180 days only.

#### Secondary analyses: specific MHCs and intensification

We then repeated the same Cox regression analysis except that we included ten binary indicator variables for the ten most prevalent specific MHCs (depressive disorders, PTSD, other anxiety disorders, adjustment disorders, psychotic disorders, bipolar disorders, substance use disorders, personality or conduct/impulse control disorders, psychogenic disorders, and other MHCs) in a single model, making it possible to examine the distinct contribution of each individual MHC in the context of any other comorbid MHCs. We estimated HRs for each of these common MHCs, for each of the three time intervals.

## Results

### Descriptive cohort characteristics

In the analytic cohort, 10,422 patients had MHC, and 42,104 had no MHC. Sample mean (SD) age was 58.3 (9.4) and 65.4 (10.6) years, respectively (p <0.001). Sample mean (SD) number of primary care visits in the 365 days before the index HbA1c was 4.3 (3.6) and 3.4 (2.7), respectively (p <0.001). Table 2 shows estimates for patient characteristics, by MHC status. The most common specific MHCs were depressive disorders (9.7%), PTSD (6.1%), other anxiety disorders (2.5%), adjustment disorders (0.5%), psychotic disorders (2.5%), bipolar disorders (1.2%), substance use disorders (2.4%), personality or conduct/impulse control disorders (0.4%), psychogenic disorders (0.2%) and all other MHCs (0.2%). Among patients with any MHC, 7,131 (68.4%) had exactly one of the 10 specific MHCs examined; others had at least two of these MHCs.

**Table 2:** **Characteristics of patients with and without mental health conditions**

	MHC Yes n = 10,422	MHC No n = 42,104
**SOCIODEMOGRAPHIC CHARACTERISTICS**		
**Age, years, %**		
<45	5.0	2.9
45-54	34.1	14.8
55-64	39.8	28.5
65-74	13.7	33.4
≥75	7.3	20.5
**Male, %**	96.4	98.5
**HEALTH STATUS**		
Physical comorbidity index, mean (SD)*	2.9 (1.9)	2.6 (1.8)
**Medical comorbidities, %** ^**†**^		
Macrovascular complications of diabetes	27.9	33.4
Ischemic heart disease	24.1	29.1
Peripheral vascular disease	6.3	7.7
Transient Ischemic Attack	0.6	0.5
Microvascular complications of diabetes	13.8	13.7
Retinopathy	3.6	4.4
Renal disease (other than end-stage renal disease)	4.8	6.3
Peripheral neuropathy	6.9	4.6
**Index HbA1c value, %**		
8.0-8.4	31.8	36.8
8.5-8.9	19.9	21.7
9.0-9.4	13.7	13.3
9.5-9.9	9.6	9.0
≥10.0	25.0	19.3

*Selim Comorbidity Index, physical component, is a count of 36 common non-psychiatric medical conditions.

^†^Comorbidities present in the 2 years prior to the Observation Interval.

*Abbreviations:* MHC, Mental Health Condition; HbA1c: Hemoglobin A1c; SD, Standard Deviation.

### Bivariate associations: MHC status and intensification or first event type

Intensification occurred within 14 days for 39.0% of patients with MHC and 37.9% of those without MHC, and within 30 days for 46.3% and 44.2%, respectively (Table 3). Intensification was the *first* event after the index high HbA1c for an estimated 64.7% and 61.0%, respectively. HbA1c <8.0 was the first event for 9.6% and 9.8%, respectively. Hospitalization was the first event for 4.3% and 2.1%, respectively; <1% of hospitalizations were for indications potentially proximately related to level of glycemic control, e.g., hospitalizations for hyperglycemia or hypoglycemia.

**Table 3:** Intensification (or alternate events) after an elevated HbA1c Value, by presence of a mental health condition, unadjusted*

Sample size	MHC Yes 10,422	MHC No 42,104
**TIME TO ANTIGLYCEMIC INTENSIFICATION**		
Intensification occurred within 14 days after high HbA1c, %	39.0	37.9
Intensification occurred within 30 days after high HbA1c, %	46.3	44.2
**FIRST EVENT AFTER INDEX HBA1C VALUE**		
First event, %		
Intensification	64.7	61.0
HbA1c value <8.0	9.6	9.8
Hospitalization	4.3	2.1
Death	0.2	0.5
No event following index HbA1c	21.2	26.5

* These unadjusted analyses are presented for descriptive purposes, so no P-values are presented.

*Abbreviations:* *MHC*, Mental Health Condition; *HbA1c*, Hemoglobin A1c.

### Main analyses: MHC status and intensification

No difference was detected in rate of intensification in the first 14 days after a high HbA1c value between patients with versus without MHC, after controlling for patient characteristics and facility (HR 0.99) (Table 4). Patients with MHC were estimated to experience a somewhat higher rate of intensification of antiglycemic medications than were those without MHC during the 15–30 and the 31–180 days following a high HbA1c (HR 1.13 and HR 1.12, respectively).

**Table 4:** Hazard ratios for antiglycemic intensification, and for the competing event, in the main model that examines MHC in aggregate*

	0-14 days		15-30 days		31-180 days	
Parameter ^†^	HR	95% CI	HR	95% CI	HR	95% CI
**MAIN ANALYSIS (Outcome: Intensification)**						
MHC (1 = yes, 0 = no)	0.99	0.96-1.03	**1.13**	1.04-1.23	**1.12**	1.07-1.18
Index HbA1c value 9–9.9^‡^	**1.24**	1.20-1.28	**1.38**	1.27-1.50	**1.36**	1.29-1.43
Index HbA1c value 10–10.9	**1.32**	1.26-1.38	**1.29**	1.15-1.45	**1.49**	1.40-1.59
Index HbA1c value 11–11.9	**1.47**	1.39-1.56	**1.68**	1.46-1.94	**1.65**	1.51-1.81
Index HbA1c value 12.0+	**1.57**	1.47-1.67	**1.47**	1.25-1.73	**1.65**	1.49-1.82
Sex (1 = male, 0 = female)	0.98	0.89-1.08	0.92	0.73-1.17	0.89	0.77-1.02
Log physical comorbidity index	1.01	0.98-1.04	1.03	0.95-1.11	**1.24**	1.18-1.30
Age (per 10 years)	**0.95**	0.94–0.97	**0.95**	0.92-0.99	**0.93**	0.91-0.95
Any macrovascular comorbidity	1.01	0.98-1.04	1.04	0.96-1.12	1.03	0.98-1.08
Any microvascular comorbidity	**0.95**	0.91-0.99	0.95	0.86-1.05	**1.15**	1.08-1.22
**COMPETING EVENT (CE) ANALYSIS (Outcome: HbA1c value <8.0)**						
MHC (CE) (1 = yes, 0 = no)					**1.16**	1.08-1.25
Index HbA1c value 9–9.9 (CE)^‡^					**0.56**	0.51-0.60
Index HbA1c value 10–10.9 (CE)					**0.31**	0.27-0.36
Index HbA1c value 11–11.9 (CE)					**0.28**	0.22-0.35
Index HbA1c value 12.0+ (CE)					**0.24**	0.18-0.32
Sex (CE) (1 = male, 0 = female)					1.08	0.86-1.35
Log physical comorbidity index (CE)					**1.29**	1.21-1.37
Age (per 10 years) (CE)					0.99	0.96-1.02
Any macrovascular comorbidity (CE)					0.95	0.89-1.01
Any microvascular comorbidity (CE)					**1.10**	1.02-1.20

***Legend:*** This table shows Hazard Ratios for intensification of antiglycemic medications in the 0–14, 15–30 and 31–180 days following HbA1c value ≥8.0, and for the competing event (HbA1c value <8.0) in the 31–180 days following HbA1c value ≥8.0.

*Hazard Ratios (HR) with statistically significant p-values (at p < .05) are shown in bold type face. Here “hazard rate” is the instantaneous rate of an event among those who have not experienced an event. The ratio of these rates under two different conditions is the “hazard ratio” (*e.g.*, with MHC versus without MHC).

^†^In the 0–14 day and 15–30 day models, hospitalization, death, subsequent HbA1c value <8.0 and end of the specific interval without an event are treated as censoring variables. In the 31–180 day model, hospitalization, death and end of the interval without an event are treated as censoring variables, and subsequent HbA1c value <8.0 is treated as a competing event (with Hazard Ratios for the competing event shown in the lower half of the table). All analyses also control for the 125 VA facilities, through 124 individual dummy variables; the hazard ratios for each of those facilities are not shown in this table, for parsimony. The overall P-value for the facilities effect is <0.001 in each of the three time intervals.

^‡^Reference group for Index HbA1c value is HbA1c value of 8–8.9.

*Abbreviations:* *MHC*, Mental Health Condition; *HbA1c*, Hemoglobin A1c; *HR*, Hazard Ratio; *CI*, Confidence Interval; *CE*, Competing Event analysis.

While not a focus of this paper, it is noteworthy that in each time interval following an elevated HbA1c value, older patients were estimated to experience a somewhat lower rate of intensification than younger patients. No independent effect of patient sex was identified, though the number of women was relatively small. Not surprisingly, patients with progressively worse glycemic control in general were estimated to experience a progressively higher rate of intensification than patients with better glycemic control.

**Figure 1:**
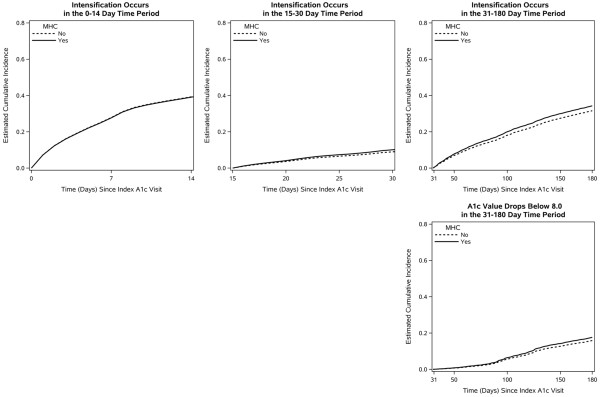
**Cumulative incidence functions for time to intensification following a high HbA1c value.** This figure shows estimated cumulative incidence functions derived from the Cox regression cause-specific hazards (adjusted for confounders at their sample mean values) for time to intensification following a high HbA1c value. Estimates are presented by Mental Health Condition status, within each of three time intervals (0–14 days, 15–30 days, 31–180 days), for the analytic cohort of diabetic patients (n =52,526). The 31–180 day cumulative incidence function is structured as a competing events analysis, with HbA1c <8.0 as a competing event. Each estimated cumulative incidence function gives the estimated cumulative probability of experiencing a particular event type (e.g., intensification) first up to that point in time. These cumulative probabilities do not reach 1 at the end of each time period (right side of each graph) due to right censoring and, during the period of 31 to 180 days, due to the concurrent accumulation of incidences of the competing event.

These patterns related to MHC were also evident in the estimated cumulative incidence functions. That is, during the later two time periods, intensification events accumulated more rapidly for those with MHC than for those with no MHC (Figure 1).

### Secondary analyses: specific MHCs and intensification

As Table 5 shows, we detected a higher rate of intensification among patients with depressive disorders at 15–30 days (HR 1.21, CI 1.08-1.36) and at 31–180 days (HR 1.12, CI 1.04-1.21), versus those with no depressive disorder. Similarly, we saw a higher rate of intensification among patients with psychogenic disorders versus those with no psychogenic disorder, at 31–180 days (HR 1.79, CI 1.21-2.64). In contrast, rate of intensification was estimated to be *lower* for patients with substance use disorders at 0–14 days (HR 0.89, CI 0.81-0.97) versus those without substance use disorders. Other differences for specific MHCs were not statistically significant.

**Table 5:** Hazard ratios for antiglycemic intensification, and for the competing event, in the secondary model that examines the distinct contribution of each of the ten specific MHCs*

	0-14 days		15-30 days		31-180 days	
Parameter ^†^	HR	95% CI	HR	95% CI	HR	95% CI
**MAIN ANALYSIS (Outcome: Intensification)**						
Depressive disorder (1 = yes, 0 = no)	1.01	0.99-1.09	**1.21**	1.08-1.36	**1.12**	1.04-1.21
PTSD	0.99	0.93-1.05	1.09	0.95-1.25	1.01	0.92-1.10
Anxiety disorder	1.01	0.92-1.11	0.89	0.71-1.12	1.10	0.97-1.26
Adjustment disorder	1.02	0.85-1.23	0.66	0.39-1.12	1.20	0.93-1.55
Psychotic disorder	0.92	0.84-1.01	1.01	0.83-1.25	1.06	0.94-1.20
Bipolar disorder	1.00	0.88-1.13	1.03	0.77-1.39	1.08	0.90-1.30
Substance use disorder	**0.89**	0.81-0.97	0.95	0.76-1.17	1.08	0.95-1.23
Personality/Impulse/Conduct disorder	0.88	0.71-1.10	1.17	0.75-1.84	0.86	0.63-1.18
Psychogenic disorder	1.27	0.95-1.69	0.88	0.39-1.97	**1.79**	1.21-2.64
Other MHC	1.15	0.86-1.53	1.49	0.80-2.79	1.03	0.64-1.64
Index HbA1c value 9–9.9‡	**1.24**	1.20-1.28	**1.38**	1.27-1.50	**1.34**	1.29-1.43
Index HbA1c value 10–10.9	**1.32**	1.26-1.38	**1.29**	1.15-1.45	**1.49**	1.40-1.59
Index HbA1c value 11–11.9	**1.48**	1.39-1.57	**1.68**	1.46-1.94	**1.65**	1.51-1.81
Index HbA1c value 12.0+	**1.57**	1.48-1.67	**1.47**	1.25-1.73	**1.65**	1.50-1.82
Sex (1 = male, 0 = female)	0.98	0.89-1.09	0.93	0.73-1.17	0.90	0.78-1.03
Log physical comorbidity index	1.00	0.97-1.04	1.03	0.96-1.11	**1.24**	1.18-1.30
Age (per 10 years)	**0.95**	0.94-0.96	**0.95**	0.92-0.99	**0.93**	0.91-0.95
Any macrovascular comorbidity	1.01	0.98-1.04	1.03	0.96-1.12	1.03	0.98-1.08
Any microvascular comorbidity	**0.95**	0.91-0.99	0.95	0.86-1.05	**1.15**	1.08-1.22
**COMPETING EVENT (CE) ANALYSIS** **(Outcome: HbA1c value <8.0)**						
Depressive disorder (CE) (1 = yes, 0 = no)					1.08	0.97-1.20
PTSD (CE)					1.01	0.89-1.14
Anxiety disorder (CE)					**1.23**	1.04-1.46
Adjustment disorder (CE)					1.39	0.99-1.94
Psychotic disorder (CE)					**1.30**	1.09-1.55
Bipolar disorder (CE)					1.20	0.93-1.55
Substance use disorder (CE)					0.98	0.80-1.20
Personality/Impulse/Conduct disorder (CE)					0.83	0.51-1.34
Psychogenic disorder (CE)					1.19	0.57-2.51
Other MHC (CE)					1.49	0.86-2.59
Index HbA1c value 9–9.9 (CE) ‡					**0.56**	0.51-0.60
Index HbA1c value 10–10.9 (CE)					**0.31**	0.27-0.36
Index HbA1c value 11–11.9 (CE)					**0.28**	0.22-0.35
Index HbA1c value 12.0+ (CE)					**0.24**	0.18-0.32
Sex (CE) (1 = male, 0 = female)					1.10	0.88-1.37
Log Physical Comorbidity Index (CE)					**1.29**	1.21-1.37
Age (per 10 years) (CE)					0.98	0.96-1.01
Any macrovascular comorbidity (CE)					0.95	0.89-1.01
Any microvascular comorbidity (CE)					**1.11**	1.02-1.20

***Legend***: This table shows Hazard Ratios for intensification of antiglycemic medications in the 0–14, 15–30 and 31–180 days following HbA1c value ≥8.0, and for the competing event (HbA1c value <8.0) in the 31–180 days following HbA1c value ≥8.0. The model includes ten binary indicator variables for the ten most prevalent specific MHCs (depressive disorders, PTSD, other anxiety disorders, adjustment disorders, psychotic disorders, bipolar disorders, substance use disorders, personality or conduct/impulse control disorders, psychogenic disorders, and other MHCs) all in a single model.

*Hazard Ratios (HR) with statistically significant p-values (at p < .05) are shown in bold face. Here “hazard rate” is the instantaneous rate of an event among those who have not experienced an event. The ratio of these rates under two different conditions is the “hazard ratio”.

^†^In the 0–14 day and 15–30 day models, hospitalization, death, subsequent HbA1c value <8.0 and end of the specific interval without an event are treated as censoring variables. In the 31–180 day model, hospitalization, death and end of the interval without an event are treated as censoring variables, and subsequent HbA1c value <8.0 is treated as a competing event (with Hazard Ratios for the competing event shown in the lower half of the table). All analyses also control for the 125 VA facilities, through 124 individual dummy variables; the hazard ratios for each of those facilities are not shown in this table, for parsimony. The overall P-value for the facilities effect is <0.001 in each of the three time intervals.

^‡^Reference group for Index HbA1c value is HbA1c value of 8–8.9.

*Abbreviations:* *MHC*, Mental Health Condition; *PTSD*, Posttraumatic stress disorder; *HbA1c*: Hemoglobin A1c; *HR*, Hazard Ratio; *CI*, Confidence Interval; *CE*, Competing Event analysis.

## Discussion

Contrary to our hypothesis, we found diabetic patients with mental health conditions were no less likely to receive intensification of antiglycemic medications in response to an elevated HbA1c value than were those without MHC. Indeed, among diabetic patients who did not receive intensification within the first two weeks following a high HbA1c value, those with MHC were marginally *more* likely than those with no MHC to receive intensification. A similar effect appeared to hold for depressive disorders and, in the 31–180 days following an elevated HbA1c value, for psychogenic disorders. In contrast, patients with substance use disorders were *less* likely than those with no substance use disorder to receive intensification in the first two weeks following a high HbA1c value, controlling for sex, age, medical comorbidity, other specific MHCs, index HbA1c value, and facility.

These findings reinforce mounting evidence that the relationship between MHC and intensity of medical care might be more complex than initially appeared. Multiple studies have concluded that patients with MHC receive less intensive medical care. This has been seen for processes and intermediate outcomes of care in a range of conditions, from cardiovascular disease [13, 31] to preventive care [32] to diabetes [9–12, 33]. Despite consistency of such findings, null effects and counter-effects have also been appearing in the literature [14, 15, 34–36].

What might account for this inconsistency? One factor may be that MHCs represent a heterogeneous group of clinically distinct conditions. Many studies aggregate MHCs, reducing ability to detect more granular effects. In studies that do distinguish between different MHCs, substance use disorders frequently emerge as drivers of low intensity care [9, 10]. Such was the case in our study as well: we estimated a lower rate of antiglycemic intensification in the first two weeks after a high HbA1c among patients with substance use disorders. While our study was not designed to identify mechanisms, this finding could reflect patient non-adherence leading clinicians to advise patients to use the currently-prescribed dose more regularly, [37] patients’ failure to attend scheduled follow-up appointments to discuss medication modifications, [38] clinician concerns about risk of hypoglycemia related to drug interactions or unreliable timing of medication intake, [39] or clinician bias leading to altered prescribing patterns [40].

Another potential contributor to observed inconsistency between studies examining medical care intensity for patients with MHC relates to differences in how care intensity was measured. Some prior studies focused on factors like level of glycemic control, which might be sensitive to patient behaviors outside the medical visit. In contrast, medication intensification, the focus of our study, may be more responsive to health care utilization patterns [36]. In our study, patients with MHC used primary care services more heavily in the year before the index HbA1c value; assuming such utilization patterns continued following the index HbA1c value, the patients with MHC might have had more opportunities to receive medication intensification. We found patients with and without MHC were equally likely to receive intensification in the first two weeks after a high HbA1c but that patients with MHC were more likely to receive intensification thereafter. This could mean that in the absence of a prompt response to a high HbA1c, clinicians are more liable to take action in patients they see more frequently. The observation that patients with more physical comorbidity were similarly more likely to receive intensification at 31–180 days – as were patients with psychogenic disorders -- bolsters this supposition.

Still another potential contributor to our finding that presence of MHC did not reduce a patient’s chances of antiglycemic intensification could relate to treatment of the MHC itself, especially in a setting like VA where mental health services are available at every medical center. The clinical presentation of patients with treated MHC (i.e., with good MHC symptom control) could resemble that of patients without MHC, attenuating differences in intensification. Indeed, treating MHC can result in improved *medical* outcomes [41].

This study has several strengths. First, it includes essentially the universe of diabetic patients on VA-prescribed oral agents with poor glycemic control, making findings more representative than would be possible in studies examining a specific setting or using primary data collection methodologies. Second, care was taken to identify sharply drawn cohorts of patients with and without MHC, reducing misclassification risk. Third, this study examines a range of MHCs and thus complements the one similar study we are aware of, which focused on depression [15]. Fourth, the longitudinal analysis approach, which accounted for competing and censoring events, allowed for more robust conclusions because the temporally dynamic nature of patient status was taken into account.

Despite these strengths, findings in this study must be interpreted subject to several caveats. First, this study used existing data. This could have led to incomplete capture of intensifications, if verbal dosage changes were not promptly recorded or if some patients received supplemental prescriptions outside VA, although VA medication pricing structures would generally make this less likely. Nonpharmacological treatments (lifestyle modification counseling) likewise would not be captured in these data, although VA encourages such counseling for all patients with diabetes. Use of existing data also could have led to under-ascertainment of MHC, [42] although VA requires routine screening for depression, PTSD and alcohol use disorder; like this study, many studies examining quality of care for patients with MHC use ICD-9-CM codes to identify MHCs [8, 31]. Models do not include an indicator for receipt of psychiatric medications, some of which can worsen glycemic control and which would therefore be expected to increase clinicians’ vigilance about monitoring glycemic state and responding to above-target HbA1c values. Severity of MHC likewise is not captured, and models do not control for patient preferences (not available in these databases). Second, our study examined intensifications. If a patient was nonadherent to a previously-prescribed medication, the provider would appropriately focus on adherence, rather than dose escalation. Third, comorbidities were set to their baseline value (present/absent) rather than treating them as time-dependent variables. Any bias thus introduced [43] should be small, since new onset of chronic conditions during the narrow OI is expected to be low. Fourth, events have been modeled as non-terminal competing events, [44] focusing on time to *first* event. This approach was appropriate to the study question examining clinical reactions proximal to an elevated HbA1c, but does not capture subsequent events. Fifth, the OI for this study ended in 2004. Diabetes management guidelines regarding target HbA1c value have evolved since then, so absolute rates of intensification in reaction to an elevated HbA1c may not entirely reflect current practice. However, the focus of this study was not upon absolute rates but rather upon *differences* in how clinicians react to patients with and without MHC, much less likely to have evolved over this time period. Sixth, the study sample came from all diabetic patients in VA who were being managed exclusively on oral antiglycemics. Because we excluded those who received any insulin during the prior six months, our cohort, while focusing on poorly-controlled diabetes, may have tended to exclude those with particularly labile glycemic control. The study sample is substantially smaller than the base cohort, but that is largely because the research question is only clinically relevant to those with elevated HbA1c. Seventh, 90% of patients with MHC and 85% of those without MHC received HbA1c testing, and thus had an opportunity to have poor glycemic control detected, if present. However, this study did not capture the relatively small group with no HbA1c testing—this may have represented patients with relatively stable diabetes (who did not need to be tested as frequently), patients not being carefully monitored, patients who did not go to the lab to complete an ordered HbA1c test, or patients receiving most of their care outside of VA. Finally, since the VA has strong systems of care for both medical and mental health conditions, study findings might not be generalizable to veterans not using VA, or to non-veterans. Results from this predominantly male sample also may not generalize to women.

## Conclusions

Our findings offer a mixed message. On one hand, it is reassuring that antiglycemic medication management appears to be at least as intensive for patients with MHC as for those without MHC, especially for depression, one of the most common MHCs in the United States [45]. On the other hand, less than half of patients received intensification in the first month after a high HbA1c. While antiglycemic intensification is sometimes clinically inappropriate even for a substantially elevated HbA1c, this study of 2003-2004 care does raise the possibility of room for performance improvement independent of mental health comorbidity status, consistent with studies documenting “clinical inertia” in many health care settings [4, 6, 46]. Furthermore, the subgroup with substance use disorders may be at risk for less intensive antiglycemic care, which may or may not be appropriate, depending on the clinical scenario. Clinicians should carefully consider the appropriate response to an elevated HbA1c value, particularly in patients with substance use disorders. Future studies should examine the potential benefits of interventions specifically targeting diabetes management in this subgroup, such as collaborative care models between primary care providers and specialty substance use disorder treatment providers, or primary care medical home models that include embedded mental health professionals [47, 48].

## Electronic supplementary material

Additional file 1: **ICD-9-CM code specifications for Mental Health Conditions.** (DOC 314 KB)

## Abbreviations

CE: Competing event analysis
CI: Confidence interval
DEpiC: Diabetes epidemiology cohorts
DSS: Decision support system
HbA1c: Hemoglobin A1c
HR: Hazard ratio
ICD-9-CM: International classification of diseases 9^th^ revision – clinical modification
MHC: Mental health condition
NPCD: National patient care database
OI: Observation interval (April 1, 2003 through March 31, 2004)
PTSD: Posttraumatic stress disorder
SD: Standard deviation
VA: Veterans health administration.

## References

[1] Institute Of Medicine Committee on Quality Health Care in America (2001). Crossing the Quality Chasm: A New Health System for the 21st Century.

[2] Kahn KL, Tisnado DM, Adams JL, Liu H, Chen WP, Hu FA, Mangione CM, Hays RD, Damberg CL (2007). Does ambulatory process of care predict health-related quality of life outcomes for patients with chronic disease?. Health Serv Res.

[3] Fleming BB, Greenfield S, Engelgau MM, Pogach LM, Clauser SB, Parrott MA (2001). The Diabetes Quality Improvement Project: moving science into health policy to gain an edge on the diabetes epidemic. Diabetes Care.

[4] McEwen LN, Bilik D, Johnson SL, Halter JB, Karter AJ, Mangione CM, Subramanian U, Waitzfelder B, Crosson JC, Herman WH (2009). Predictors and impact of intensification of antihyperglycemic therapy in type 2 diabetes: translating research into action for diabetes (TRIAD). Diabetes Care.

[5] **Intensive blood-glucose control with sulphonylureas or insulin compared with conventional treatment and risk of complications in patients with type 2 diabetes (UKPDS 33). UK Prospective Diabetes Study (UKPDS) Group** *Lancet* 1998,**352**(9131)**:**837–853. 9742976

[6] Phillips LS, Branch WT, Cook CB, Doyle JP, El-Kebbi IM, Gallina DL, Miller CD, Ziemer DC, Barnes CS (2001). Clinical inertia. Ann Intern Med.

[7] Pogach LM, Tiwari A, Maney M, Rajan M, Miller DR, Aron D (2007). Should mitigating comorbidities be considered in assessing healthcare plan performance in achieving optimal glycemic control?. Am J Manag Care.

[8] Redelmeier DA, Tan SH, Booth GL (1998). The treatment of unrelated disorders in patients with chronic medical diseases. N Engl J Med.

[9] Frayne SM, Halanych JH, Miller DR, Wang F, Lin H, Pogach L, Sharkansky EJ, Keane TM, Skinner KM, Rosen CS, Berlowitz DR (2005). Disparities in diabetes care: impact of mental illness. Arch Intern Med.

[10] Desai MM, Rosenheck RA, Druss BG, Perlin JB (2002). Mental disorders and quality of diabetes care in the Veterans Health Administration. Am J Psychiatry.

[11] Jones LE, Clarke W, Carney CP (2004). Receipt of diabetes services by insured adults with and without claims for mental disorders. Med Care.

[12] Goldberg RW, Kreyenbuhl JA, Medoff DR, Dickerson FB, Wohlheiter K, Fang LJ, Brown CH, Dixon LB (2007). Quality of diabetes care among adults with serious mental illness. Psychiatr Serv.

[13] Mitchell AJ, Lord O, Malone D (2012). Differences in the prescribing of medication for physical disorders in individuals with v. without mental illness: meta-analysis. Br J Psychiatry.

[14] Weiss AP, Henderson DC, Weilburg JB, Goff DC, Meigs JB, Cagliero E, Grant RW (2006). Treatment of cardiac risk factors among patients with schizophrenia and diabetes. Psychiatr Serv.

[15] Katon W, Russo J, Lin EH, Heckbert SR, Karter AJ, Williams LH, Ciechanowski P, Ludman E, Von Korff M (2009). Diabetes and poor disease control: is comorbid depression associated with poor medication adherence or lack of treatment intensification?. Psychosom Med.

[16] Lustman PJ, Anderson RJ, Freedland KE, de Groot M, Carney RM, Clouse RE (2000). Depression and poor glycemic control: a meta-analytic review of the literature. Diabetes Care.

[17] Hankin CS, Spiro A, Miller DR, Kazis L (1999). Mental disorders and mental health treatment among U.S. Department of Veterans Affairs outpatients: the Veterans Health Study. Am J Psychiatry.

[18] Kazis LE, Miller DR, Clark J, Skinner K, Lee A, Rogers W, Spiro A, Payne S, Fincke G, Selim A, Linzer M (1998). Health-related quality of life in patients served by the Department of Veterans Affairs: results from the Veterans Health Study. Arch Intern Med.

[19] Frayne SM, Parker VA, Christiansen CL, Loveland S, Seaver MR, Kazis LE, Skinner KM (2006). Health Status Among 28,000 Women Veterans. The VA Women’s Health Program Evaluation Project. J Gen Intern Med.

[20] Frayne SM, Phibbs CS, Saechao F, Maisel NC, Friedman SA, Finlay A, Berg E, Balasubramanian V, Dally SK, Ananth L, Romodan Y, Lee J, Iqbal S, Hayes PM, Zephyrin L, Whitehead A, Torgal A, Katon JG, Haskell S (2014). Sociodemographics, Utilization, Costs of Care, and Health Profile, Volume 3. Sourcebook: Women Veterans in the Veterans Health Administration.

[21] Seal KH, Metzler TJ, Gima KS, Bertenthal D, Maguen S, Marmar CR (2009). Trends and risk factors for mental health diagnoses among Iraq and Afghanistan veterans using Department of Veterans Affairs health care, 2002–2008. Am J Public Health.

[22] Miller DR, Safford MM, Pogach LM (2004). Who has diabetes? Best estimates of diabetes prevalence in the Department of Veterans Affairs based on computerized patient data. Diabetes Care.

[23] Jha AK, Perlin JB, Kizer KW, Dudley RA (2003). Effect of the transformation of the Veterans Affairs Health Care System on the quality of care. N Engl J Med.

[24] Shah BR, Hux JE, Laupacis A, Zinman B, van Walraven C (2005). Clinical inertia in response to inadequate glycemic control: do specialists differ from primary care physicians?. Diabetes Care.

[25] Pogach LM, Brietzke SA, Cowan CL, Conlin P, Walder DJ, Sawin CT (2004). Development of evidence-based clinical practice guidelines for diabetes: the Department of Veterans Affairs/Department of Defense guidelines initiative. Diabetes Care.

[26] Frayne SM, Miller DR, Sharkansky EJ, Jackson VW, Wang F, Halanych JH, Berlowitz DR, Kader B, Rosen CS, Keane TM (2010). Using administrative data to identify mental illness: what approach is best?. Am J Med Qual.

[27] Putter H, Fiocco M, Geskus RB (2007). Tutorial in biostatistics: competing risks and multi-state models. Stat Med.

[28] Selim A, Fincke G, Ren X, Lee A, Rogers W, Miller D, Linzer M, Kazis L, Goldfield N, Pine M, Pine J (2002). The Comorbidity Index. Measuring and Managing Health Care Quality, Volume 4.

[29] Harrell FE (2010). Regression Modeling Strategies: with application to linear models, logistic regression, and survival analysis.

[30] Rosthoj S, Andersen PK, Abildstrom SZ (2004). SAS macros for estimation of the cumulative incidence functions based on a Cox regression model for competing risks survival data. Comput Methods Programs Biomed.

[31] Druss BG, Bradford DW, Rosenheck RA, Radford MJ, Krumholz HM (2000). Mental disorders and use of cardiovascular procedures after myocardial infarction. JAMA.

[32] Druss BG, Rosenheck RA, Desai MM, Perlin JB (2002). Quality of preventive medical care for patients with mental disorders. Med Care.

[33] Williams LH, Miller DR, Fincke G, Lafrance JP, Etzioni R, Maynard C, Raugi GJ, Reiber GE (2010). Depression and incident lower limb amputations in veterans with diabetes. J Diabetes Complications.

[34] Petersen LA, Normand SL, Druss BG, Rosenheck RA (2003). Process of care and outcome after acute myocardial infarction for patients with mental illness in the VA health care system: are there disparities?. Health Serv Res.

[35] Brown CH, Medoff D, Dickerson FB, Kreyenbuhl JA, Goldberg RW, Fang L, Dixon LB (2011). Long-term glucose control among type 2 diabetes patients with and without serious mental illness. J Nerv Ment Dis.

[36] Krein SL, Bingham CR, McCarthy JF, Mitchinson A, Payes J, Valenstein M (2006). Diabetes Treatment Among VA Patients With Comorbid Serious Mental Illness. Psychiatr Serv.

[37] Heisler M, Hogan MM, Hofer TP, Schmittdiel JA, Pladevall M, Kerr EA (2008). When more is not better: treatment intensification among hypertensive patients with poor medication adherence. Circulation.

[38] Arici C, Ripamonti D, Maggiolo F, Rizzi M, Finazzi MG, Pezzotti P, Suter F (2002). Factors associated with the failure of HIV-positive persons to return for scheduled medical visits. HIV Clin Trials.

[39] *VA/DOD Clinical Practice Guideline for the Management of Diabetes Mellitus, Version 4.0*. [http://www.healthquality.va.gov/]

[40] Hall JA, Roter DL, Milburn MA, Daltroy LH (1996). Patients’ health as a predictor of physician and patient behavior in medical visits. A synthesis of four studies. Med Care.

[41] Lustman PJ, Griffith LS, Freedland KE, Kissel SS, Clouse RE (1998). Cognitive behavior therapy for depression in type 2 diabetes mellitus. A randomized, controlled trial. Ann Intern Med.

[42] Perez-Stable EJ, Miranda J, Munoz RF, Ying YW (1990). Depression in medical outpatients. Underrecognition and misdiagnosis. Arch Intern Med.

[43] van Walraven C, Davis D, Forster AJ, Wells GA (2004). Time-dependent bias was common in survival analyses published in leading clinical journals. J Clin Epidemiol.

[44] Fine JP, Jiang H, Chappell R (2001). On Semi-Competing Risks Data. Biometrika.

[45] Kessler RC, Berglund P, Demler O, Jin R, Merikangas KR, Walters EE (2005). Lifetime prevalence and age-of-onset distributions of DSM-IV disorders in the National Comorbidity Survey Replication. Arch Gen Psychiatry.

[46] Berlowitz DR, Ash AS, Hickey EC, Friedman RH, Glickman M, Kader B, Moskowitz MA (1998). Inadequate management of blood pressure in a hypertensive population. N Engl J Med.

[47] Sullivan G, Han X, Moore S, Kotrla K (2006). Disparities in Hospitalization for Diabetes Among Persons With and Without Co-occurring Mental Disorders. Psychiatr Serv.

[48] Keyser DJ, Houtsinger JK, Watkins K, Pincus HA (2008). Applying the institute of medicine quality chasm framework to improving health care for mental and substance use conditions. Psychiatr Clin North Am.

[CR49] The pre-publication history for this paper can be accessed here:http://www.biomedcentral.com/1472-6963/14/458/prepub

