# Supplementary material for: Mental illness and intensification of diabetes medications: an observational cohort study
Source: BMC Health Serv Res. 2014 Oct 22;14:458. doi: 10.1186/1472-6963-14-458 (PMC4282515; doi:10.1186/1472-6963-14-458)
Supplement: Supplementary file 1 — Additional file 1: ICD-9-CM code specifications for Mental Health Conditions. (DOC 314 KB) [file 12913_2014_3573_MOESM1_ESM.doc]

**Additional file 1: ICD-9-CM code specifications for Mental Health Conditions**

|  | **ICD-9-CM** |
| --- | --- |
| **Depressive disorders** |  |
|  | 29620 |
|  | 29621 |
|  | 29622 |
|  | 29623 |
|  | 29624 |
|  | 29625 |
|  | 29626 |
|  | 29630 |
|  | 29631 |
|  | 29632 |
|  | 29633 |
|  | 29634 |
|  | 29635 |
|  | 29636 |
|  | 3004 |
|  | 311 |
|  |  |
| **PTSD** |  |
|  | 30981 |
|  |  |
| **Other anxiety disorders** |  |
|  | 30000 |
|  | 30001 |
|  | 30002 |
|  | 30009 |
|  | 30010 |
|  | 30020 |
|  | 30021 |
|  | 30022 |
|  | 30023 |
|  | 30029 |
|  | 3003 |
|  |  |
| **Adjustment disorders** |  |
|  | 3090 |
|  | 3091 |
|  | 30922 |
|  | 30923 |
|  | 30924 |
|  | 30928 |
|  | 30929 |
|  | 3093 |
|  | 3094 |
|  | 30982 |
|  | 30983 |
|  | 30989 |
|  | 3099 |
|  |  |
| **Psychotic disorders** |  |
|  | 29500 |
|  | 29501 |
|  | 29502 |
|  | 29503 |
|  | 29504 |
|  | 29505 |
|  | 29510 |
|  | 29511 |
|  | 29512 |
|  | 29513 |
|  | 29514 |
|  | 29515 |
|  | 29520 |
|  | 29521 |
|  | 29522 |
|  | 29523 |
|  | 29524 |
|  | 29525 |
|  | 29530 |
|  | 29531 |
|  | 29532 |
|  | 29533 |
|  | 29534 |
|  | 29535 |
|  | 29540 |
|  | 29541 |
|  | 29542 |
|  | 29543 |
|  | 29544 |
|  | 29545 |
|  | 29550 |
|  | 29551 |
|  | 29552 |
|  | 29553 |
|  | 29554 |
|  | 29555 |
|  | 29560 |
|  | 29561 |
|  | 29562 |
|  | 29563 |
|  | 29564 |
|  | 29565 |
|  | 29570 |
|  | 29571 |
|  | 29572 |
|  | 29573 |
|  | 29574 |
|  | 29575 |
|  | 29580 |
|  | 29581 |
|  | 29582 |
|  | 29583 |
|  | 29584 |
|  | 29585 |
|  | 29590 |
|  | 29591 |
|  | 29592 |
|  | 29593 |
|  | 29594 |
|  | 29595 |
|  | 2970 |
|  | 2971 |
|  | 2972 |
|  | 2973 |
|  | 2978 |
|  | 2979 |
|  | 2980 |
|  | 2981 |
|  | 2983 |
|  | 2984 |
|  | 2988 |
|  | 2989 |
|  |  |
| **Bipolar disorders** |  |
|  | 29600 |
|  | 29601 |
|  | 29602 |
|  | 29603 |
|  | 29604 |
|  | 29605 |
|  | 29606 |
|  | 29610 |
|  | 29611 |
|  | 29612 |
|  | 29613 |
|  | 29614 |
|  | 29615 |
|  | 29616 |
|  | 29640 |
|  | 29641 |
|  | 29642 |
|  | 29643 |
|  | 29644 |
|  | 29645 |
|  | 29646 |
|  | 29650 |
|  | 29651 |
|  | 29652 |
|  | 29653 |
|  | 29654 |
|  | 29655 |
|  | 29656 |
|  | 29660 |
|  | 29661 |
|  | 29662 |
|  | 29663 |
|  | 29664 |
|  | 29665 |
|  | 29666 |
|  | 2967 |
|  | 29680 |
|  | 29681 |
|  | 29682 |
|  | 29689 |
|  |  |
| **Substance use disorders** |  |
|  | 29182 |
|  | 2910 |
|  | 2913 |
|  | 2914 |
|  | 2915 |
|  | 2918 |
|  | 29181 |
|  | 29189 |
|  | 2919 |
|  | 30300 |
|  | 30301 |
|  | 30302 |
|  | 30303 |
|  | 30390 |
|  | 30391 |
|  | 30392 |
|  | 30393 |
|  | 30500 |
|  | 30501 |
|  | 30502 |
|  | 30503 |
|  | 29285 |
|  | 64830 |
|  | 64831 |
|  | 64832 |
|  | 64833 |
|  | 64834 |
|  | 96500 |
|  | 96501 |
|  | 96502 |
|  | 96509 |
|  | 2920 |
|  | 29211 |
|  | 29212 |
|  | 2922 |
|  | 29281 |
|  | 29283 |
|  | 29284 |
|  | 29289 |
|  | 2929 |
|  | 30400 |
|  | 30401 |
|  | 30402 |
|  | 30403 |
|  | 30410 |
|  | 30411 |
|  | 30412 |
|  | 30413 |
|  | 30420 |
|  | 30421 |
|  | 30422 |
|  | 30423 |
|  | 30430 |
|  | 30431 |
|  | 30432 |
|  | 30433 |
|  | 30440 |
|  | 30441 |
|  | 30442 |
|  | 30443 |
|  | 30450 |
|  | 30451 |
|  | 30452 |
|  | 30453 |
|  | 30460 |
|  | 30461 |
|  | 30462 |
|  | 30463 |
|  | 30470 |
|  | 30471 |
|  | 30472 |
|  | 30473 |
|  | 30480 |
|  | 30481 |
|  | 30482 |
|  | 30483 |
|  | 30490 |
|  | 30491 |
|  | 30492 |
|  | 30493 |
|  | 30520 |
|  | 30521 |
|  | 30522 |
|  | 30523 |
|  | 30530 |
|  | 30531 |
|  | 30532 |
|  | 30533 |
|  | 30540 |
|  | 30541 |
|  | 30542 |
|  | 30543 |
|  | 30550 |
|  | 30551 |
|  | 30552 |
|  | 30553 |
|  | 30560 |
|  | 30561 |
|  | 30562 |
|  | 30563 |
|  | 30570 |
|  | 30571 |
|  | 30572 |
|  | 30573 |
|  | 30580 |
|  | 30581 |
|  | 30582 |
|  | 30583 |
|  | 30590 |
|  | 30591 |
|  | 30592 |
|  | 30593 |
|  |  |
| **Personality disorders and conduct/impulse control disorders** |  |
|  | 3010 |
|  | 30110 |
|  | 30111 |
|  | 30112 |
|  | 30113 |
|  | 30120 |
|  | 30121 |
|  | 30122 |
|  | 3013 |
|  | 3014 |
|  | 30150 |
|  | 30159 |
|  | 3016 |
|  | 3017 |
|  | 30181 |
|  | 30182 |
|  | 30183 |
|  | 30184 |
|  | 30189 |
|  | 3019 |
|  |  |
|  | 3128 |
|  | 31200 |
|  | 31201 |
|  | 31202 |
|  | 31203 |
|  | 31210 |
|  | 31211 |
|  | 31212 |
|  | 31213 |
|  | 31220 |
|  | 31221 |
|  | 31222 |
|  | 31223 |
|  | 3124 |
|  | 31281 |
|  | 31282 |
|  | 31289 |
|  | 3129 |
|  | 31381 |
|  | 31230 |
|  | 31231 |
|  | 31232 |
|  | 31233 |
|  | 31234 |
|  | 31235 |
|  | 31239 |
|  |  |
| **Psychogenic disorders** |  |
|  | 3060 |
|  | 3061 |
|  | 3062 |
|  | 3063 |
|  | 3064 |
|  | 30650 |
|  | 30652 |
|  | 30653 |
|  | 30659 |
|  | 3066 |
|  | 3067 |
|  | 3068 |
|  | 3069 |
|  | 30011 |
|  | 3007 |
|  | 30081 |
|  | 30082 |
|  | 30780 |
|  | 30789 |
|  | 30270 |
|  | 30276 |
|  | 30279 |
|  | 30289 |
|  | 3029 |
|  | 30651 |
|  | 30016 |
|  | 30019 |
|  | 3005 |
|  | 30753 |
|  | 30754 |
|  | 30151 |
|  |  |
| **Other mental health conditions** |  |
|  | 3071 |
|  | 30750 |
|  | 30751 |
|  | 30759 |
|  | 30012 |
|  | 30013 |
|  | 30014 |
|  | 30015 |
|  | 3006 |
|  | 3080 |
|  | 3081 |
|  | 3082 |
|  | 3083 |
|  | 3084 |
|  | 3089 |
|  | 64840 |
|  | 64841 |
|  | 64842 |
|  | 64843 |
|  | 64844 |
|  | 316 |
|  | 30089 |
|  | 3009 |
|  | 2982 |
|  | 29690 |
|  | 29699 |

***Legend***: This table shows the ICD-9-CM codes mapping to each of the ten specific mental health conditions (MHCs): depressive disorders, posttraumatic stress disorder (PTSD), other anxiety disorders, adjustment disorders, psychotic disorders, bipolar disorders, substance use disorders, personality or conduct/impulse control disorders, psychogenic disorders and other MHCs. Any digits after the first three digits of the ICD-9-CM code represent digits following a decimal point.
